# Effect of vitamin D supplementation on assisted reproduction technology (ART) outcomes and underlying biological mechanisms: protocol of a randomized clinical controlled trial. The “supplementation of vitamin D and reproductive outcome” (SUNDRO) study

**DOI:** 10.1186/s12884-019-2538-6

**Published:** 2019-11-01

**Authors:** Alessio Paffoni, Edgardo Somigliana, Veronica Sarais, Stefania Ferrari, Marco Reschini, Sofia Makieva, Enrico Papaleo, Paola Viganò

**Affiliations:** 1 Obstetrics and Gynaecology Department, Fondazione IRCCS Ca’ Granda Ospedale Maggiore Policlinico, via M. Fanti 6, 20122 Milan, Italy; 2 Dipartimento di Scienze Cliniche e di Comunità, University of Milan, via Fanti 6, 20122 Milan, Italy; 3 Obstetrics and Gynaecology Department, IRCCS San Raffaele Scientific Institute, via Olgettina 60, 20132 Milan, Italy; 4 Division of Genetics and Cell Biology, Reproductive Sciences Laboratory, IRCCS San Raffaele Scientific Institute, via Olgettina 60, 20132 Milan, Italy

**Keywords:** IVF, Infertility, Vitamin D, 25(OH)D, Pregnancy rate, Cumulus cells, Endometrium, Oocyte quality, Follicular fluid

## Abstract

**Background:**

Vitamin D plays an important role in human physiology and pathology. The receptor for vitamin D regulates 0.5–5% of the human genome. Accordingly, vitamin D insufficiency has been shown to increase the risk of several diseases. In recent years, based on growing evidence, on a role of vitamin D has been also postulated in reproductive health both in animals and humans, especially in female fertility female fertility. In vitro fertilization success was shown to be higher in women with appropriate reserves of vitamin D. However a causal relation has not been demonstrated and randomized controlled trials testing the effectiveness of vitamin D supplementation in IVF are warranted.

**Methods:**

This is a multicenter randomized double blinded placebo controlled study aimed at determining the benefits of vitamin D [25(OH)D] supplementation in improving clinical pregnancy rate in women undergoing IVF. Eligible women with a serum level of 25-hydroxyvitamin D [25(OH)D] < 30 ng/ml will be randomized. Recruited women will be given the drug (either 600,000 IU of 25(OH) D or placebo in a single oral administration) at the time of randomization. Two centres will participate and the sample size (700 women) is foreseen to be equally distributed between the two. Patients will be treated according to standard IVF protocols.

**Discussion:**

The primary aim of the study is the cumulative clinical pregnancy rate per oocyte retrieval. Clinical pregnancy is defined as the presence of at least one intrauterine gestational sac with viable foetus at first ultrasound assessment (3 weeks after a positive human chorionic gonadotropin [hCG] assessment). Secondary outcomes include: 1) clinical and embryological variables; 2) oocyte and endometrium quality at a molecular level. To investigate this latter aspect, samples of cumulus cells, follicular and endometrial fluids will be obtained from a subgroup of 50 age-matched good-prognosis cases and controls.

**Trial registration:**

The protocol was included in EudraCT on 22nd September 2015 with the registration number assigned ‘2015-004233-27’; it was submitted through the database of the Italian “Osservatorio Nazionale della Sperimentazione Clinica (OsSC)” - (National Monitoring Centre of Clinical Trials) to the National Competent Authority on 8th March 2016 and approved on 23rd June 2016.

## Background

Vitamin D plays an important role in human physiology and pathology. The activation of the vitamin D receptor (VDR) may regulate directly and/or indirectly the expression of a very large number of genes (0.5–5% of the total human genome). Accordingly, vitamin D insufficiency has been shown to increase the risk of several diseases including cancers, auto-immune diseases, infections and pregnancy complications [1, 2].

In recent years, a role of vitamin D has been also postulated in reproductive health. VDR is expressed in the ovary, the endometrium and the myometrium [3]. Moreover, vitamin D has been shown to influence female fertility potential in animals and humans [4, 5]. In vitro fertilization (IVF) success was demonstrated to be higher in women with appropriate reserves of vitamin D [6]. In a recent contribution from our group, we detected an adjusted odds ratio (a-OR) (adjusted for age, ethnicity, BMI, parity, duration of infertility, number of retrieved oocytes, number of transferred embryos, and study period) of 2.15 [95% confidence interval (CI): 1.23–3.77] for clinical pregnancy rate in women with vitamin D > 20 ng/ml compared to those with vitamin D < 20 ng/ml. A similar figure was observed when considering the implantation rate. Of note, this beneficial effect was even more evident when using a threshold of 30 ng/ml [7]. The large body of evidence available in animal models and data from in vitro studies in women strongly favour a causal relation, but evidence from RCTs is required to definitely support a role for vitamin D in influencing the chances of pregnancy. To date, only a small RCT has been published and no benefits emerged [8]. However, the study was insufficiently powered to draw definite conclusions (in total 117 women). Moreover, only women scheduled for frozen embryo transfer were included.

Exploring the mechanisms at the basis of the beneficial effects of vitamin D could also be relevant and may open new avenues of research. To date, there are few appealing findings but the scenario is incomplete, especially from interventional studies. Biological evidence from RCTs could conversely provide crucial insights on the role of vitamin D in influencing oocytes quality or endometrial receptivity.

Oocytes cannot be directly tested for molecular analyses. However, the evaluation of the cumulus and granulosa cells of the ovarian follicle could indirectly reveal the quality of the corresponding oocytes. The gene expression profile of cumulus-oocyte-complexes has been suggested to predict which embryos have the best chance of implanting in the uterus. In particular, the expression of COX20, BMP15 and GDF9 has been associated with the establishment of pregnancy or live birth. Other possible potential genes of interest but with less consistency among studies include GREM1, HAS2, VCAN, PTGS [9, 10].

Endometrial samples can conversely be obtained prior to initiate the IVF cycle and can thus be directly studied without interfering with the procedure. Of interest here is that the endometrium and the early pregnancy decidua also express VDR and could be other important targets of vitamin D. In a previous study from our group, vitamin D was shown to modulate the expression of genes important for embryo receptivity and to interact with the local cytokines in human endometrial cells [11].

In the present study the following hypotheses will be tested: 1) 25-hydroxyvitamin D [25(OH)D] deficiency negatively affects pregnancy rates in IVF cycles with an effect mediated through endometrial receptivity and/or oocyte embryo quality; 2) Vitamin D supplementation and restoration of adequate vitamin D levels may actually improve the prognosis of infertile women undergoing IVF procedures. The absolute magnitude of the benefit deemed to be clinically relevant in terms of cumulative clinical pregnancy rate is considered 10% (from 20 to 30%); 3) The molecular profiles of endometrium, follicular fluid and cumulus cells are modified in vitamin D-supplemented women with respect to molecules known to be associated with IVF outcomes and embryo implantation process.

## Methods

The study has been designed according to the CONSORT methodology.

### Aim

The present trial is aimed at determining the benefits of vitamin D supplementation in improving cumulative clinical pregnancy rate in women undergoing IVF. In particular, the three main aims of the study are as follows:

- Aim 1: To determine the potential benefits of vitamin D supplementation in improving cumulative clinical pregnancy rate in women undergoing IVF.
- Aim 2: To investigate whether vitamin D supplementation might improve clinical and embryological variables such as: number of cancelled cycles, number of available oocytes, number of good quality embryos, units of FSH administered per retrieved oocyte, implantation rate of transferred embryos.
- Aim 3: To investigate the effect of vitamin D supplementation on oocyte and endometrium quality at a molecular level through a comparative analysis between treated and untreated patients based on the analysis of cumulus cells, follicular and endometrial fluids.

### Setting

This is a multicenter randomized superiority double blinded placebo controlled clinical trial with parallel groups and allocation 1:1.

Two Italian academic infertility units will be involved:

- IRCCS Fondazione Ca’ Granda, Ospedale Maggiore Policlinico, Milan, Italy.
- IRCCS Ospedale San Raffaele, Milan, Italy.

### Participants

Inclusion criteria of women will be as follows: indication to IVF, age 18–39 years, less than 3 previous IVF attempts, body mass index (BMI) 18–25 kg/m^2^.

Exclusion criteria will be as follows: patients with anti-mullerian hormone serum level < 0.5 ng/ml, IVF cycle using frozen embryos/oocytes, vitamin D contraindications/side effects for consumption of vitamin D (high blood calcium levels or conditions that can lead to high calcium levels such as sarcoidosis, tuberculosis, parathyroid disease).

Eligible women will be informed on the study design and those agreeing to participate will sign an informed consent and will provide a blood sample for 25(OH) D assessment. Those with a serum level < 30 ng/ml will be randomized.

A CONSORT flow diagram of the study protocol is provided as an additional figure (see Additional file 1).

### Interventions

#### Experimental design aim 1

Recruited women will be given the drug (either 600,000 IU of 25-OH-vitamin D or placebo in a single oral administration) at the time of randomization, thus 2–12 weeks prior to oocyte retrieval and managed according to a standardized clinical protocol of ovarian stimulation with in vitro fertilization (fresh cycle). Administration of the drug/placebo will be performed by the physician in order to guarantee a compete adherence to the protocol. No dose changes are foreseen. Patients will be asked to avoid further consumption of vitamin D after randomization.

Women whose cycle will be cancelled will not be excluded (intention to treat analysis). A specific software will establish the randomization list. Sun exposure during the study period will be recorded but it will not be a reason for drop-out. Serum 25-OH-Vitamin D will be reassessed at the time of hCG administration or, if the cycle will be cancelled, at the time of the decision to interrupt the treatment. The quantitative detection of total serum 25(OH) D levels will be performed using a commercially available kit based on a chemiluminescence technology (DiaSorin) with intra- and interassay coefficients of variations equal to 10 and 15%, respectively.

Patients will be treated according to standard IVF protocols as described elsewhere [12, 13]: since neither the clinicians nor the recruited women will be aware about vitamin D dosage, clinical management of ovarian stimulation will not be conditioned by the study.

In case of supernumerary oocytes or embryos deriving from the fresh cycle, the subsequent embryo transfer using thawed embryos or oocytes will be considered for the study outcome if performed within 4 months from oocyte retrieval and 25(OH) D serum levels will be reassessed.

Serum hCG assessment to detect pregnancy will be performed at + 14/+ 16 days after hCG administration. If positive, women will undergo a transvaginal sonography 3 weeks later.

Clinical pregnancy (primary outcome) is defined as the presence of at least one intrauterine gestational sac with viable foetus.

Dates of the study: 27/06/2016–26/06/2019

**Fig. 1:**
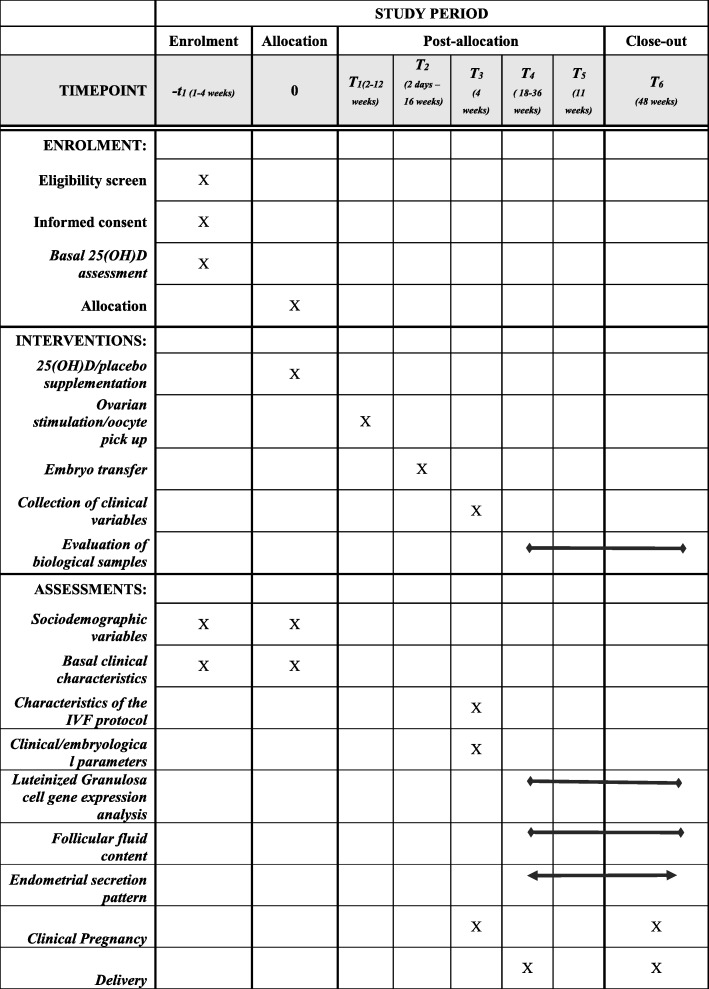
Schedule of enrolment, interventions and assessments of the SUNDRO trial

A schedule of enrolment, interventions and assessments of the study protocol is reported in Fig. 1.

#### Experimental design aim 2

In order to investigate whether vitamin D supplementation might improve clinical and embryological parameters, we will use our electronic dataset to compare with appropriate statistical tests all of the variables that are routinely collected in IVF cycles. The main variables that we will consider in order to discover possible effects of vitamin D supplementation on the clinical outcome of the IVF cycle are: rate of cancelled cycles, number of available oocytes, units of FSH administered per retrieved oocyte (they are related to the rate of appropriate response of the patients to the stimulation protocol), number of good quality embryos on the day of embryo transfer as evaluated according to the Istanbul Consensus (it is related with the quality of gametes) [14], implantation rate of transferred embryos (it is related to the quality of embryos and endometrium), delivery rate and obstetric outcomes.

#### Experimental design aim 3

A subgroup (*n* = 50) of good-prognosis cases and controls will be recruited among randomized patients for collection and analysis of biological samples as described below.

##### Luteinized granulosa cell gene expression analysis

Human luteinized granulosa cells (GC) from placebo- and vitamin D- supplemented patient groups will be isolated from the discarded follicular fluid post oocyte retrieval according to standard protocols [15]. Briefly, cells in follicular fluid will be isolated by initial centrifugation of the pool of follicular fluid (2000×g for 10 min) followed by gradient separation at density of 1.077 g/mL (Lymphoprep™, STEMCELL Technologies, Vancouver, Canada). Following the gradient centrifugation (1700×g for 20 min), three layers will emerge: a top layer containing the follicular fluid, a bottom layer containing erythrocytes and the middle layer containing mononuclear cells. The mononuclear middle layer will be collected and supplemented with 3 ml of RPMI 1640 medium containing 10% fetal bovine serum. To eliminate the macrophages, the mononuclear cell suspension will be incubated in a Petri plate, at 37 °C for 15 min. It is expected that, while macrophages will adhere to the plastic during this time frame, luteinized GCs and lymphocytes will not. An additional step to separate GCs from lymphocytes will be performed by maintaining the cell suspension for 24 h at 37 °C under standard culture conditions. Due to their physical properties, lymphocytes, in contrast to GCs, cannot attach to plastic, thus, allowing an effective segregation of the two cell types. GCs will be lysed using the RLT buffer (QIAGEN) and total RNA will be sentd for RNAseq analysis. TruSeq Stranded Total RNA Library Prep Kit will be used for library preparation and sequenced on an Illumina HiSeq2500 Rapid Run System, generating pair-end 50 base-pair reads (140 M reads on average per sample). Transcript abundance will be estimated with Kallisto [16] and differentially expressed (DE) genes will be identified using DeSeq2 R package [17] and a corrected *p*-value < 0.05. The DE genes that will emerge from RNAseq analysis between placebo and vitamin D-supplemented groups, will be validated with RT-PCR using SYBR green system, QIAGEN pre-validated, ready-to-use primer sets and QIAGEN real-time PCR cycler, the Rotor-Gene Q. A new cohort of samples will be employed for the validation. Western blotting analysis will be used to confirm the changes in gene expression at the protein level.

##### Follicular fluid content

BMP-15 and GDF-9 are currently regarded as the best candidate molecules responsible for oocyte regulation of cumulus cell expansion during ovulation. Follicular samples from both groups of women will be retrieved via transvaginal route under ultrasound guidance. Follicular fluids will be centrifuged at 1000×g for 10 min to remove cellular components; the clear supernatant will be divided into aliquots and frozen at − 80 °C until further use. Prior to processing, the follicular fluids will be centrifuged and filtered on Millipore filter to remove precipitates. A chemiluminescent assay will be used to compare BMP-15, GDF-9 and 25(OH) D levels in follicular fluid of women treated or not with vitamin D supplementation.

##### Endometrial secretion pattern

Endometrial secretions from both groups of women treated or not with vitamin D supplementation will be aspirated using a catheter during the secretory phase of the menstrual cycle prior to frozen embryo transfer: a multiplex immunoassay will be performed to compare the cytokine profile. Interleukin (IL)-1ß, IL-10, IL-12, IL-15, IL-17, tumor necrosis factor (TNF)-α, eotaxin, glycodelin A, granulocyte colony-stimulating factor, monocyte chemotactic protein-1, vascular endothelial growth factor, interferon-gamma will be analysed given their known involvement in human embryo implantation.

### Data collection and analysis

An electronic dataset will be filled (SPSS ver. 20, IBM).

All data will be recorded with paper case report forms and entered electronically at both participating sites using a common dataset. Original study forms will be stored in numerical order and stored in a secure and accessible place for 30 years after study completion. A biostatistician will perform quality control before data analysis, including check for double data entry and range for data values. Missing data will be included in the analysis using specific modern imputation methods.

Statistically significant differences will be determined using Fisher’s exact test, chi-squared test, Student’s t test or the Wilcoxon test, as appropriate.

An interim analysis (315 randomized completed cycles) of clinical pregnancy rates will be performed in order to highlight unforeseen or higher-than-expected effects of vitamin D supplementation. According to the Haybittle–Peto boundary rule, the statistical significance for deciding to stop the trial is set to *p*-value < 0.001 [18]. Results of the interim analysis will be available to all medical and laboratory staff. The steering committee decides on the continuation of the trial and will report to the involved ethics committees.

In accordance with national guidelines, the main outcome will be collected for every woman in order to complete their clinical charts. Since the main outcome is the clinical pregnancy rate as (defined as the presence of at least one intrauterine gestational sac with viable foetus), it has to be noted that its evaluation is highly standardized and fully objective. Moreover, according to legal requirements and standard clinical protocols, secondary outcomes related to the evolution of pregnancies will be easily collected through usual methods and reminders for contacting patients.

Given the study design, protocol adherence is foreseen to be complete. However, data will be analysed according to the “intention to treat” analysis.

Since the clinical trial is designed as minimal risk a formal committee for data monitoring is not foreseen.

Electronic monitoring of the datasets will be performed by two independent physicians every 3 months during recruitment focussing on: completeness of the data, eligibility criteria, date of randomization, treatment assignment, adverse events and endpoints, signed informed consent.

### Sample size calculation

The sample size was calculated according to Kane [19] based on the following assumptions:

1) the primary outcome is the cumulative clinical pregnancy rate (CPR) per started cycle; 2) expected CPR in the deficient population 20% [7], 3) expected CPR in the supplemented population 30% [7], 4) expected proportion of women with insufficient serum vitamin D (< 30 ng/ml): 90% [20], 5) Type I and II errors of 0.05 and 0.20, respectively. Under these assumptions, a sample size of 300 randomized women per arm will allow us to detect with sufficient statistical power (*p* < 0.05) an absolute difference ≥ 10% in the CPR rate between the two groups using the chi-squared test.

Considering that 10% of recruited women are expected to have serum vitamin D levels ≥30 ng/ml (therefore they will not be randomized) and considering a dropout rate of 5%, it has been decided to recruit 700 women to reach the number of 600 required subjects. The full formula used for sample size calculation is provided as an additional figure (see Additional file 2).

### Randomization

Randomization will be organized centrally by UOC Farmacia of the Fondazione IRCCS Ospedale Maggiore Policlinico. The allocation sequence will be computer-generated and hidden from the participants as well as from the physicians and biologists involved in the clinical management of the patients. The allocation ratio will be 1:1. Randomization list will be stratified for the two participating centres. Since the random list has not other stratification, the allocation sequence is considered unpredictable and no restrictions are planned in order to reduce predictability.

### Blinding

All patients, all caregivers and embryologists at the clinical departments will be blinded to trial intervention allocation. This is possible because ampoules of drug/placebo will be prepared and coded in a undistinguishable way by the local central pharmacy of the Fondazione IRCCS Ca′ Granda according to a computer generated random list. The pharmacy will reveal the content of administered ampoule only after cycle completion.

The allocation sequence will be implemented through administration of anonymized ampoules in the progressive order they are coded by the pharmacy.

The progress of the study will be periodically monitored by an external monitor in order to verify the strictness of the data management. The medical doctors evaluating the main outcome will also be blinded to the trial intervention allocation. Unblinding will be possible after the embryo transfer procedure. In case of adverse effects of vitamin D supplementation, as reported by participating women, the care giver of the patient can obtain information on the participant’s allocated intervention asking to the principal investigator who will ask the pharmacy using a specific module.

### Consent

Women eligible for the study will be referred by care providers to a member of the research team who will describe the informed consent process. Details of the study will be explained to patients, including its main aims, procedures, temporal commitment, possible discomforts and risks, benefits. Recruitment will be on a voluntary basis with the right to withdraw from the study at any time; moreover, women will be informed that the decision to join the study protocol will not affect their future treatments. Volunteers will sign the informed consent after giving them the opportunity to put questions and enough time to consider their participation.

### Confidentiality

Recruited patients will be assigned anonymous codes and data analysis will be performed on anonymized datasets. Patient data collected during the study protocol will be treated in accordance with the Italian 196/2003 Data Protection Act. Electronic datasets will be accessed through a personal password; paper documentation will be accessible only to study investigators. Study data will be kept for at least 10 years after publication of study results.

## Discussion

For the most part, vitamin D is derived from the conversion of pro-vitamin D under the essential effect of solar ultraviolets. This particular mean of actually satisfying our needs represents an important concern. The mutated habits of humans in Western Worlds is not compatible with an adequate exposure to sunlight and thus hamper the provision of sufficient vitamin D [2].

The effect of vitamin D supplementation in women belonging to infertile couples has been insufficiently explored yet, although several groups have highlighted better chances of pregnancy in women replete for serum vitamin D [6]. Therefore, the hypothesis that vitamin D supplementation could improve IVF results deserves further consideration.

The present study can potentially influence the clinical practice of infertility treatment. Vitamin D insufficiency can be simply overcome. Supplementation with vitamin D is economic, simple and effective and would represent an innovative and simple adjuvant treatment of IVF with the potential additional benefit of improving the chances of natural conception. Of further relevance here is that vitamin D supplementation is free of relevant side effects.

Dosage of supplementation was based on previous data showing that a single 600.000 IU dose of cholecalciferol is able to rapidly increase serum 25(OH) D levels and to maintain adequate levels up to 90 days after administration in young subjects, including patients with severe vitamin D depletion. The proposed dosage is indeed considered safe since it gives peak levels well below the widely accepted toxic blood levels of 200 ng/ml [21, 22]. Of note, the single dose administered in-office by the physician guarantees a total adherence to treatment. However, given the lack of causal relationship between vitamin D levels and IVF outcomes and the risk of excessive vitamin D serum levels, patients will be asked to avoid further consumption of vitamin D after randomization. Moreover, at a molecular level, our analysis can provide preliminary data on reproductive cells that can be influenced by circulating vitamin D levels (endometrium, follicle, oocyte cumulus-oocyte complex). Whether the effect of vitamin D in human reproduction is mediated at ovarian, endometrial or both levels represents a matter of controversy. According to our prospective cross-sectional study aiming at investigating IVF outcomes in women with deficient 25(OH) D level, the number of top quality embryos and, as a consequence, the rate of women reaching the stage of blastocyst transfer was higher in women with sufficient levels of serum 25(OH)D. The chances of embryo implantation were also enhanced supporting the idea that the impact of the compound might be exerted in both the reproductive tissues [7]. Disentangling the potential role of vitamin D in the endometrium is particularly relevant given the key role of uterine factors in determining the outcome of IVF treatment, the significant gaps in our knowledge of mechanisms underlying endometrial-embryo asynchrony and most of all, the lack of relevant modifiers of endometrial receptivity status [23]. Also of particular note, there is growing and consistent evidence that vitamin D supplementation may improve birth outcomes and prevent some relevant obstetrics complications. According to a recent systematic review of the randomized studies on vitamin D supplementation, the compound increases mean birth weight of 58.33 g (95%CI 18.88–97.78 g) [24]. Moreover, a lower risk in pre-eclampsia was observed in those women receiving vitamin D supplementation, especially if they received also calcium [25].

Some limitations of our study should be acknowledged. Firstly, it has to be pointed out that the decision for a single dose of 600.000 IU is a pragmatic choice and remarkably simplifies our study design. Moreover, if significant benefits will emerge, this modality could easily enter clinical practice (considering also the low costs) and a high adherence can be expected. On the other hand, a non-significant result will not be a definitive reason to abandon the studies on vitamin D supplementation prior to IVF because one could argue that a more steady and constant administration (such as 2.000 IU daily) could be more beneficial. Secondly, the duration of vitamin D supplementation prior to IVF can be an additional reason of concern, in particular considering that we allowed for a certain variability between Vitamin D administration and the initiation of the cycle (2–12 weeks). In fact, folliculogenesis has been claimed to last 5–6 months and our protocol does not allow us to entirely cover this long period. Our protocol actually consents to properly test the effects of vitamin D on endometrial growth (a menstrual flow will systematically occur after the administration) and on final follicular growth (from dominance to ovulation) but not on the full folliculogenesis process. Again, we decided to privilege pragmatism and simplicity with the aim of facilitating future clinical use but, again, we have to admit that this choice can expose our results to some criticisms if no significant difference will emerge. Thirdly, we will request recruited women to avoid vitamin D consumption but we cannot request them to avoid sun exposure during the whole period of the IVF cycle. Even if one has to expect a similar frequency of this situation in the two study arms, this potential confounder might partly dilute the potential benefits of the tested supplementation of vitamin D. On the other hand, again, we opted for a pragmatic study and this confounder would just reflect real life situations. Fourthly, the decision to focus on women with insufficient vitamin D (< 30 ng/ml) rather than deficient (< 20 ng/ml) or even severely deficient (< 10 ng/ml) could be argued. In fact, the study will be underpowered for a reliable subgroup analysis according to the magnitude of the deficiency. In other words, we will not be able to assess whether the intervention could be of value only in a selected group (in particular those with the lowest levels) rather than for the whole heterogeneous group of women with serum levels below 30 ng/ml. Fifthly, the decision to randomize even women with serum levels below 10 ng/ml could be ethically debatable because this is a condition of possible clinical relevance per se. However, it has to be underlined that there is currently no recommendation for vitamin D testing before IVF and, for this reason, we did not deem unethical to include all women with serum levels below 30 ng/ml.

Overall, despite the above-mentioned limitations of our study design, we believe that this RCT could provide clinically relevant findings, in particular if statistically significant differences in favour of the treatment arm will emerge. In fact, it can influence clinical practice. High doses supplementation with vitamin D is an extremely simple and safe intervention and, if a significant benefit will emerge, one has to expect a rapid and global spread of its use. In addition, given the low costs of the intervention (less than 10 € in Italy), the economical profile can be expected to be highly cost-effective. On the other hand, for the above-mentioned intrinsic limitations consequent to our pragmatic choices, we have to recognize that a negative finding will not definitely close the issue.

## Supplementary information

**Additional file 1.** CONSORT flow diagram of the SUNDRO study.

**Additional file 2.** Full formula for sample size calculation.

## Data Availability

The datasets used and analysed during the current study will be available from the corresponding author on reasonable request.

## Abbreviations

25(OH)D: 25-hydroxyvitamin D
a-OR: Adjusted odds ratio
ART: Assisted Reproduction Technology
CI: 95% confidence interval
CPR: Clinical pregnancy rate
IVF: In vitro fertilization
RCTs: Randomized controlled trials
SUNDRO: Supplementation of vitamin D and reproductive outcome
VDR: Vitamin D receptor
