# Supplementary material for: Effect of vitamin D supplementation on assisted reproduction technology (ART) outcomes and underlying biological mechanisms: protocol of a randomized clinical controlled trial. The “supplementation of vitamin D and reproductive outcome” (SUNDRO) study
Source: BMC Pregnancy Childbirth. 2019 Nov 1;19:395. doi: 10.1186/s12884-019-2538-6 (PMC6823943; doi:10.1186/s12884-019-2538-6)
Supplement: Supplementary file 1 — Additional file 1. CONSORT flow diagram of the SUNDRO study. [file 12884_2019_2538_MOESM1_ESM.doc]

**
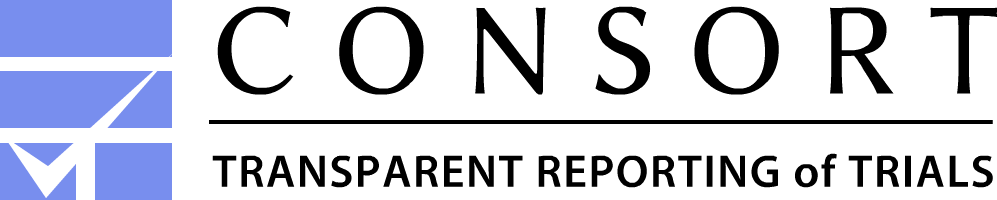
**

**CONSORT 2010 Flow Diagram**

**Allocation**

**Analysis**

**Follow-Up**

**Enrollment**

Expected to be assessed for eligibility (n=700)

Expected to be excluded (n=70, 10% of assessed women)

♦  Vitamin D ≥30ng/ml (n=70)

Planned to analyse (n=300, 95% of randomized women)

Expected to lose to follow-up (no information regarding birth, n=15, 5% of randomized women)

Expected to be allocated to intervention (n=315, 50% of randomized women)

♦ Expected to receive Vitamin D (n=315)

Expected to lose to follow-up (no information regarding birth, n=15, 5% of randomized women)

Expected to be allocated to intervention (n=315, 50% of randomized women)

♦ Expected to receive Placebo (n=315)

Planned to analyse (n=300, 95% of randomized women)

Expected to randomize (n=630, 90% of assessed women)
