# Supplementary figures and images for: Effect of vitamin D supplementation on assisted reproduction technology (ART) outcomes and underlying biological mechanisms: protocol of a randomized clinical controlled trial. The “supplementation of vitamin D and reproductive outcome” (SUNDRO) study
Source: BMC Pregnancy Childbirth. 2019 Nov 1;19:395. doi: 10.1186/s12884-019-2538-6 (PMC6823943; doi:10.1186/s12884-019-2538-6)

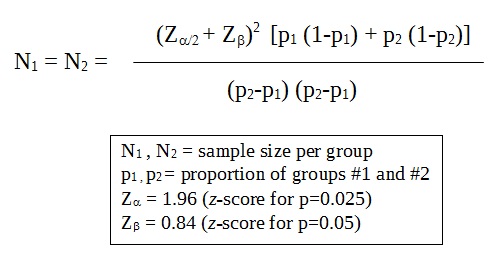

Supplement: Supplementary file 2 — Additional file 2. Full formula for sample size calculation. [file 12884_2019_2538_MOESM2_ESM.jpg]
